# Balanced Hybrid Nutrient Density Score Compared to Nutri-Score and Health Star Rating Using Receiver Operating Characteristic Curve Analyses

**DOI:** 10.3389/fnut.2022.867096

**Published:** 2022-05-02

**Authors:** Adam Drewnowski, Tanhia D. Gonzalez, Colin D. Rehm

**Affiliations:** ^1^Center for Public Health Nutrition, University of Washington, Seattle, WA, United States; ^2^PepsiCo, Inc., Plano, TX, United States; ^3^PepsiCo, Inc., Purchase, NY, United States

**Keywords:** nutrient profiling, HEI-2015, Health Star Rating, receiver operating characteristic (ROC), Nutri-score

## Abstract

**Background:**

Nutrient profiling (NP) models that are used to assess the nutrient density of foods can be based on a combination of key nutrients and desirable food groups.

**Objective:**

To compare the diagnostic accuracy of a new balanced hybrid nutrient density score (bHNDS) to Nutri-Score and Health Star Rating (HSR) front-of-pack systems using receiver operating characteristic (ROC) curve analyses. The diet-level bHNDS was first validated against Healthy Eating Index (HEI-2015) using data from the 2017–18 National Health and Nutrition Examination Survey (2017–18 NHANES). Food-level bHNDS values were then compared to both the Nutri-Score and HSR using ROC curve analyses.

**Results:**

The bHNDS was based on 6 nutrients to encourage (protein, fiber, calcium, iron, potassium, and vitamin D); 5 food groups to encourage (whole grains, nuts and seeds, dairy, vegetables, and fruit), and 3 nutrients (saturated fat, added sugar, and sodium) to limit. The algorithm balanced components to encourage against those to limit. Diet-level bHNDS values correlated well with HEI-2015 (*r* = 0.67; *p* < 0.001). Food-level correlations with both Nutri-Score (*r* = 0.60) and with HSR (*r* = 0.58) were significant (both *p* < 0.001). ROC estimates of the Area Under the Curve (AUC) showed high agreement between bHNDS values and optimal Nutri-Score and HSR ratings (>0.90 in most cases). ROC analysis identified those bHNDS cut-off points that were predictive of A-grade Nutri-Score or 5-star HSR. Those cut-off points were highly category-specific.

**Conclusion:**

The new bHNDS model showed high agreement with two front-of-pack labeling systems. Cross-model comparisons based on ROC curve analyses are the first step toward harmonization of proliferating NP methods that aim to “diagnose” high nutrient-density foods.

## Introduction

The main purpose of nutrient profiling (NP) models is to identify foods of high nutritional value (1, 2) in order to promote adherence to dietary guidelines (3, 4). Without forgetting the need to restrict saturated fat, added sugar, and salt, the US Dietary Guidelines for Americans (DGA) call for healthy food patterns that feature whole grains, legumes, nuts and seeds, dairy products, vegetables, and fruits (3–5). The same shift toward food-based dietary guidelines has occurred globally (6). Consistent with global trends, current NP methods to assess the healthfulness of foods (7) are no longer purely nutrient-based but have expanded to include dietary ingredients and desirable food groups (7, 8).

Many NP models have become the basis of front-of-pack labeling in the European Union and elsewhere (1, 2) and assessing their diagnostic accuracy is a public health priority, given that many such models can produce disparate results (1, 2). NP model components can include both nutrients and selected food groups to encourage as well as nutrients or food groups to limit (8–10). In one such model (8), nutrients to encourage were protein, fiber, potassium, calcium, iron, and vitamin D (8). Food groups to encourage were whole grains, nuts, seeds, dairy products, vegetables, and fruits (8), whereas nutrients to limit were saturated fat, added sugar, and sodium. Many NP models are compensatory in the sense that the positive elements (nutrients and food groups) compensate for the nutrients to limit. However, NP models are not necessarily balanced, placing more weight on the positive than on negative elements or vice versa (8, 11).

This study had two aims. The first aim was to validate a new balanced hybrid NP model against an independent measure of a healthy diet. Following past studies (12), we compared diet-level balanced hybrid nutrient density score (bHNDS) values with Healthy Eating Index (HEI-2015) scores for the same participants in the 2017—18 NHANES database. The second aim was to compare the diagnostic accuracy of alternative NP models using receiver operating characteristic (ROC) curve analyses (13, 14). The three NP models were the new bHNDS model, the French Nutri-Score (15, 16), and the Health Star Rating (HSR) from Australia-New Zealand (17). Each of these models was specifically designed to identify foods of high nutritional value, albeit in very different ways. Nutri-Score and the HSR treat dietary energy as an element to limit (15–17), whereas the Nutrient Rich Food (NRF) family of scores does not (8). Both Nutri-Score and the HSR are partly compensatory, with positive nutrients or food groups unable to compensate for excess calories, fat, sugar, or salt. The HSR is partly category-specific since it places dairy products in separate categories with more relaxed scoring criteria (17).

Food companies are beginning to screen product portfolios using multiple NP models. Those models do not necessarily assign foods to the same categories. ROC analyses can be used to establish NP values that are predictive of the sought-after A-grade Nutri-Score or 5-star HSR front-of-pack ratings. Cross-model comparisons based on ROC curve analyses of sensitivity, specificity, and area under the curve (AUC) can assist both regulatory agencies and the food industry in selecting the best methods to identify nutrient-dense foods. Having a test for the diagnostic accuracy of different NP models would be the first step toward the potential harmonization of the front-of-pack labels.

## Materials and Methods

### Food and Nutrient Data and Inclusion Criteria

Data on energy content and nutrient composition of foods came from the United States Department of Agriculture (USDA) Food and Nutrient Database for Dietary Studies (FNDDS 2017–18) (18), which is used to assess dietary intakes in 2017–18 NHANES. FNDDS provides energy and nutrient values for 7,083 foods and beverages. All foods are classified and coded into food groups, subgroups, and categories using the What We Eat In America (WWEIA) 1-digit, 2-digit, and 4-digit coding systems (19). The FNDDS database was merged with the USDA Food Patterns Equivalents Database (FPED) and Food Patterns Ingredient Database (FPID) to estimate amounts of desirable food ingredients in mixed foods (20).

This analysis excluded human milk, baby foods, infant formula, low-energy-density foods/beverages (<5 kcal/100 g), sugars, honey, protein and nutritional powders, alcoholic drinks, water and enhanced water, unprocessed meats, poultry, or seafood (other than frozen, dried, canned, or ready-to-eat products), mixed dishes (other than frozen, canned, or ready-to-eat products), and items not coded as foods. The Nutri-Score and HSR are not applicable for these product categories. Low-energy-density diet beverages, water, unsweetened coffee, or tea were also excluded since the nutrient density of foods is often defined as the ratio of nutrients to calories (12, 21).

Unprepared forms of foods were used for cooked grains and similar products (rice, oats, and pasta). Both Nutri-Score and the HSR are calculated per 100 g of unprepared dry product. Of the 3,377 eligible foods, 2,723 were consumed by NHANES 2017–18 participants on their first recall day. Consumption frequency weighted analyses ensured that frequently eaten foods had a greater impact on the analyses than infrequently eaten foods.

### The Healthy Eating Index

Dietary intake data for assessments of dietary quality came from the first in-person 24-h recall completed by participants aged ≥ 2 y in the NHANES 2017–18 (22, 23). All participants completed a 24-h dietary recall, reporting all foods/beverages consumed from midnight to midnight before the data collection date. Depending on their age, children’s reports were completed by caregivers (for ages < 6 y), or the child completed the recall with the assistance of a parent/guardian (age 6–11 y) (22, 23). Staff from the National Center for Health Statistics assessed all recalls for plausibility. All data used in the present research is publicly available on the USDA and CDC websites and are completely de-identified. As such, this research is not considered human subjects research, and no ethical approvals were sought (22). The National Center for Health Statistics has obtained ethical approval for NHANES (23).

Diet quality of NHANES 2017–18 participants aged ≥ 2 y (*n* = 7,122) was assessed using the HEI-2015 (24), a measure of adherence to each successive edition of the Dietary Guidelines for Americans (DGA). The HEI-2015 is an energy-adjusted diet quality score with a range of 0 (low adherence) to 100 (high adherence) that includes 13 components: 9 components to encourage and 4 components to limit (24). Components to encourage include total fruit, whole fruit, total vegetables, beans and greens, whole grains, dairy, total protein, seafood/plant protein, and the ratio of unsaturated to saturated fats. The limiting components are refined grains, sodium, saturated fat, and added sugars. To calculate the HEI-2015, dietary intake data from NHANES 2017–18 was merged with the USDA Food Patterns Equivalents Database (FPED) (20).

### Characteristics of the Balanced Hybrid Nutrient Density Score Model

Dietary fiber, calcium, potassium, and vitamin D were identified by the DGA as nutrients of public health concern (3, 4). The DGA also recommended food patterns featuring whole grains, nuts and seeds, vegetables, fruit, and low-fat dairy (5). The so-called “hybrid” NP models of nutrient density have used a combination of nutrients and food groups. The initial nutrient-based NRF 9.3 model was based on 9 nutrients to encourage and 3 nutrients to limit (12, 21). The later hybrid NRFh 6:5:3 model (8) was based on 6 nutrients and five food groups to encourage and the same 3 nutrients to limit (8). The choice of score elements was dictated by the DGA (3, 4).

Model algorithms were based on percent daily values, that is ratios of the nutrient content of food relative to the nutrient daily value per reference amount. In this case, 100 kcal was the reference amount. Each component of the bHNDS was thus expressed as a percent of the daily value (%DV) calculated per 100 kcal of food. Following past procedures, %DV was capped at 100% so that foods with very large amounts of a single nutrient would not get an overly highly total score (12, 21). The sub-score based on the mean of %DV for 6 nutrients and 5 food groups to encourage was defined as ENC11. The negative sub-score based on the mean of %DV (Or %MRV, maximum recommended values) for 3 nutrients to limit was LIM3. The final algorithm was bHNDS = ENC11-LIM3.

The present bHNDS model contained an important modification. The bHNDS positive and negative subscores were the means of %DV rather than the sums of %DV. The net effect of this approach, previously used in the French SAIN, LIM model (11) but not in NRF9.3 (12, 21) was to weight the overall score more heavily toward the negative components. Effectively, summed %DVs for saturated fat added sugar, and sodium was divided by 3, whereas summed %DVs for the positive elements were divided by 11. The nutrients and food groups that were included in the bHNDS model are shown in Table 1, together with their reference values. The components of the Balanced Hybrid Nutrient Density (bHNDS) scores are given as follows:

**TABLE 1:** Components of the NRFh6:5:3 nutrient profiling model and reference values.

bHNDS components	Nutrients to encourage	Reference value^a^
ENC11	Protein^#^	50 g
	Fiber^#^	28 g
	Calcium^#^	1,300 mg
	Iron^#^	18 mg
	Potassium^#^	4,700 mg
	Vitamin D^#^	20 μg
	Food groups to encourage	
	Vegetables + legumes	2.5 cup equiv
	Fruit	2.0 cup equiv
	Whole grains	3.0 oz equiv
	Dairy	3.0 cup equiv
	Nuts and seeds	0.75 oz equiv^b^
	Nutrients to limit	
LIM3	Added sugars^#^	50 g (11.9 tsp equiv)
	Sodium^#^	2,300 mg
	Saturated fat^#^	20 g

*^a^Reference values for nutrients sourced from FDA and for food groups from USDA MyPlate Recommendations using the mode of age/sex-specific recommendations. ^b^The USDA does not provide a specific recommendation for nuts/seeds. We used 0.75 oz/equiv which corresponds to approximately 1.5 ounces a recommendation from the American Heart Association ^#^Flagged factors are included in the NRF9.3. The original NRF9.3 did not include vitamin D, but later iterations have replaced vitamin E, with vitamin D.*

**Center of the circle:** $\text{bHNDS}=\bar{\text{ENC11}}-\bar{\text{LIM3}}$


ENC11¯=∑i=111Nutrients⁢and⁢Food⁢Groups⁢to⁢Encourage⁢per⁢ 100⁢kcaliReference⁢Valuei11

LIM3¯=∑i=13Nutrients⁢to⁢Limit⁢per⁢ 100⁢kcaliReference⁢Valuei3

Abbreviations:

$\bar{E\,N\,C\,11}$ = Average of Nutrients and Food Groups to Encourage

$\bar{L\,I\,M\,3}$ = Average of Nutrients to Limit

### Nutri-Score and Health Star Rating

The FSA-Ofcom 2004 score developed in the pre-Brexit UK became the basis of Nutri-Score in France (13) and the HSR in Australia and New Zealand (14). Relevant details for calculating the two scores are described in detail elsewhere (17, 25). Both Nutri-Score and HSR use a negative sub-score that is based on foods’ energy density, and amounts of saturated fat, total sugar, and sodium per 100 g or milliliters. Both models also used a positive sub-score that is based on protein, fiber, and the foods content (% weight) of fruits, nuts, vegetables, and legumes (FNVL). Since ingredient-level data is not available for all foods in FNDDS, the % weight of FNVL was estimated based on servings of these food groups from FPED. Nutri-Score and HSR values were calculated for all eligible FNDDS foods and beverages, including fresh vegetables and fruits.

For front-of-pack labels for solid foods, Nutri-Score points are converted into color-coded letter grades: scores ≤ −1 translate to A, scores 0–2 become B, scores 3–10 become C, scores 11–18 become D, and scores ≥ 19 become E (25). Beverages in Nutri-Score are treated differently but also receive letter grades. Similarly, HSR ratings are converted to a star scoring system (17).

### Comparisons of Diet-Level Balanced Hybrid Nutrient Density Score and Nutrient Rich Food 9.3 Scores With Healthy Eating Index-2015

Following past procedures (12, 21), diet-level bHNDS and NRF9.3 scores were applied to the total diets of participants in the NHANES 2017–18 sample (age ≥ 2 y). Dietary nutrient density was calculated per 2,000 kcal to be consistent with the HEI-2015 scores, which are also calculated on a per calorie basis. Regression analyses were conducted using HEI-2015 as the dependent variable and the diet-level bHNDS and NRF9.3 scores as the independent variables. All the models were adjusted for gender, ethnicity, and age. All the analyses were weighted using the NHANES sample weights and adjusted for the complex sample design of NHANES. These analyses were conducted for the entire NHANES population and by age groups (2–9 y, 10–19 y, 20–39 y, 40–64 y, and ≥ 65 y).

### Receiver Operating Characteristic and Area Under the Curve Analyses

Receiver operating characteristic (ROC) analyses were used to assess the ability of the bHNDS to discriminate foods by their Nutri-Score and HSR values. ROC curve analyses are commonly used to evaluate the accuracy of diagnostic tests (16, 17). We first calculated the frequency of consumption-weighted correlations between the raw Nutri-Score and HSR values and the bHNDS. Violin plots were then used to compare the distribution of bHNDS scores for each Nutri-Score and HSR value. Significant trends were identified using a weighted linear regression model.

Thresholds for the ROC analysis were A and A/B for Nutri-Score and 5 stars and ≥ 4.5 stars for the HSR. ROC analyses plotted the sensitivity (true positive rate) and 1-specificity (false positive rate) at each value of bHNDS and then calculated the AUC, which can range from 0 to 1.0, with higher values indicating the greater predictive ability of the bHNDS. An AUC value of 0.5 is equivalent to random allocation. While some have proposed rules for interpreting AUC values (e.g., >0.9 indicating excellent agreement), there is emerging consensus that these guidelines are reductive and should not be used.

Whenever bHNDS values are generally predictive of Nutri-Score or HSR values, an algorithmically defined optimal cut-off value can be identified. The identification of this value can depend on multiple factors, including the pros/cons of false positives vs. false negatives. Some algorithms have been put forward to aid in the choice of optimal cut-off values, and we opted to use the method of Liu (26), which refers to the point along the ROC curve that maximizes the product of sensitivity and specificity. Additional analyses also evaluated other approaches, such as the Youden Index, which chooses the cut-off point based on the sum of sensitivity and specificity (27), but the results were generally identical or very similar. Because of the differences in the distribution of bHNDS values by food category, ROC analyses were conducted across all food and beverages and by 24 modified WWEIA food categories.

All analyses were conducted using Stata 16.0 (College Station, TX) and were weighted to incorporate survey weights, NHANES analyses, and the weighted frequency of consumption for food-level analyses.

## Results

### The Balanced Hybrid Nutrient Density Score Characteristics

The bHNDS model is fully compensatory, meaning that the positive subscore is balanced 50:50 against the negative subscore. In theory, bHNDS scores can range from −100 to 100; the observed range was −46 to 61.

One concern about NP models that combine both nutrients and food groups is the potential co-linearity of model components. A correlation matrix was constructed (Supplementary Table 1) to better understand the inter-relations among bHNDS components. The expected associations between fiber, vegetables, and legumes and between dairy, calcium, and vitamin D were observed. However, there was no relation between whole grains and the fiber content of foods, and no co-linearity was observed. The inclusion of desirable food groups in the NP algorithm did not diminish the importance of nutrients (28).

### Correlations Between Diet-Level Balanced Hybrid Nutrient Density Score and Healthy Eating Index-2015 in National Health and Nutrition Examination Survey 2017–18 Database

**FIGURE 1:**
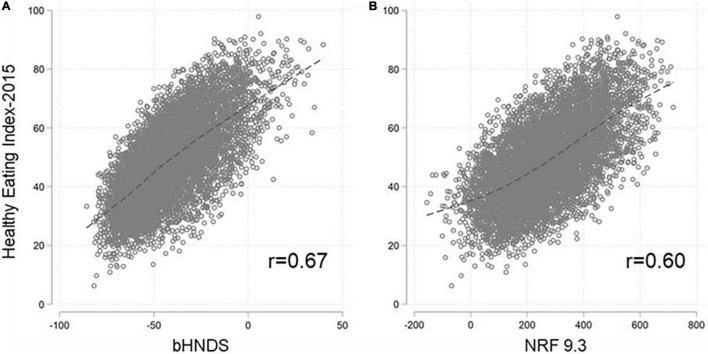
Relation between bHNDS and HEI-2015 **(A)** and between NRF 9.3 and HEI-2015 **(B)**.

The correlation between the bHNDS and HEI-2015 scores for 2017–2018 NHANES participants (Figure 1) was *r* = 0.67, a value close to the previous reports of another hybrid NP model (10). There was a modest improvement over the original NRF 9.3 nutrient-based model (*r* = 0.60). The correlations between bHNDS and HEI-2015 held for the total NHANES population and across age groups. Age-specific analyses were necessary to assess NP model performance across age groups; age is the most profound determinant of differences in dietary intakes. The corresponding correlations by age were as follows: 0.7 (2–9 y); 0.66 (10–19 y); 0.65 (20–39 y); 0.65 (40–64 y); and 0.73 (≥65 y).

### Associations and Correspondence Between Balanced Hybrid Nutrient Density Score, Nutri-Score, and Health Star Rating Raw Scores

**FIGURE 2:**
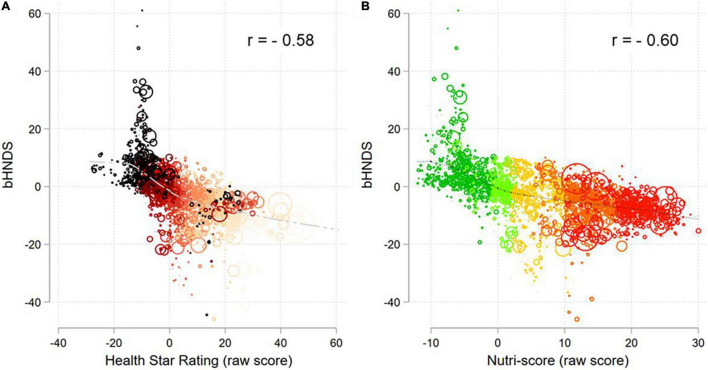
Association between bHNDS and Health Star Rating **(A)** and Nutri-Score raw scores **(B)**.

Figure 2 shows the relation between bHNDS, Nutri-Score, and HSR for the entire FNDDS database (*n* = 2,723). The relationship was inverse since both Nutri-Score and HSR assign fewer scores to more nutrient-dense foods whereas bHNDS and other models do the opposite. The size of the circle corresponds to the number of times the item was consumed by NHANES participants. The correlation between bHNDS and the other two systems was −0.60 for Nutri-Score and −0.58 for HSR.

### Associations and Correspondence Between Balanced Hybrid Nutrient Density Score, Nutri-Score Grades, and Health Star Rating Stars

**FIGURE 3:**
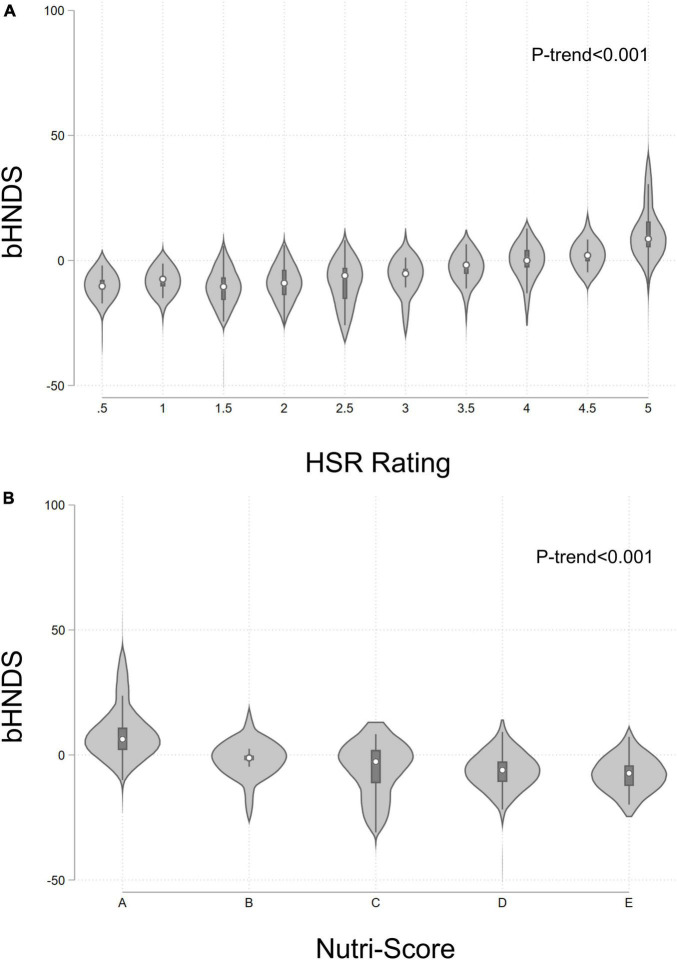
Distribution of bHNDS scores by HSR **(A)** and Nutri-Score **(B)** values.

Figure 3 shows the distribution of bHNDS values by Nutri-Score and HSR values using violin plots (26). Violin plots are similar to box plots in showing the group median (white dot), interquartile range (black bar), and the lower and upper adjacent values. Violin plots also show the probability density of the data at different values. Data in Figure 3 shows a direct relation between bHNDS values, Nutri-Score letter grades, and HSR stars. The plot also shows substantial heterogeneity of bHNDS scores at each level of the other scores. Tests for trends were conducted using a weighted linear regression model treating HSR and Nutri-Score as continuous variables (*p* < 0.001 for both).

### Receiver Operating Characteristic Analyses by Food Category

Results for ROC analyses are shown in Tables 2A,B. Data are presented for all foods and by food category. There was a high degree of correspondence between bHNDS and Nutri-Score, with AUC values > 0.88 for all four criteria, indicating a high level of agreement. The data indicate that bHNDS is generally predictive of whether food will get high HSR or Nutri-Score ratings.

**TABLE 2A:** Performance of bHNDS against Health Star Rating and Nutri-Score by food category.

	bHNDS		HSR ≥ 4.5			N-S A|B		
	N	Group mean (SD)	%	AUC	bHNDS cut-off point^a^	%	AUC	bHNDS cut-off point^a^
All			31.5	0.93		35.4	0.88	

Vegetables	390	13.7 (12.9)	90.9	0.97	1.24	97.2	0.94	1.24
Fruit	110	7.2 (4.0)	93.1	0.97	5.11	97.7	0.99	−2.94
Nuts/seeds	77	7.7 (2.8)	62.4	0.98	7.59	56.3	0.98	7.88
100% juice	41	4.8 (3.0)	0.0	−	−	5.0	0.26	6.63
Milk and milk substitutes	84	2.2 (4.5)	88.4	0.94	−0.34	4.9	1.00	12.29
Beans, peas, and legumes	63	1.6 (3.6)	83.5	0.99	−1.57	99.7	0.71	0.21
Cooked grains	26	1.3 (1.9)	2.9	0.97	4.68	92.1	0.31	−
Breakfast cereals	148	0.5 (4.0)	26.3	0.99	3.95	31.1	0.97	3.35
Potatoes	85	−0.9 (1.7)	51.8	0.95	−0.87	69.0	0.99	−1.27
Breads, rolls and tortillas	145	−0.9 (2.1)	10.1	0.89	1.38	68.9	0.91	−1.84
Yogurt	31	−1.0 (2.3)	70.8	0.66	−1.49	96.0	0.74	−1.94
Processed soy products	13	−1.6 (6.2)	7.3	0.77	−0.03	71.7	0.99	−2.87
Snacks	224	−2.0 (2.3)	1.6	0.95	0.89	8.9	0.74	−1.19
Meat, poultry and seafood	14	−2.2 (4.7)	1.2	0.99	2.51	32.1	1.00	−2.65
Quick breads and bread products	100	−4.7 (2.3)	0.0	−	−	4.2	0.97	−2.38
Mixed dishes	132	−4.8 (4.7)	10.4	0.84	−1.34	51.3	0.62	−3.15
Cheese	62	−5.7 (1.7)	23.6	0.89	−5.04	3.3	0.95	−3.86
Sweets and desserts	456	−8.1 (3.3)	0.1	0.98	−0.62	2.7	0.76	−2.59
Fats and oils	98	−8.4 (3.7)	4.2	0.29	−	0.0	−	−
Processed meat	102	−9.2 (2.3)	0.0	−	−	0.0	−	−
Jams, syrups and toppings	33	−11.7 (2.7)	1.1	1.00	1.19	1.2	1.00	−8.32
Coffee and tea	58	−12.3 (5.7)	0.0	−	−	1.4	0.78	−10.33
Sweetened beverages	109	−13.7 (6.5)	0.0	−	−	2.1	0.94	−6.90
Condiments and sauces	122	−14 (10.9)	12.9	1.00	−2.29	38.8	0.87	−20.54

*^a^The cut-off point is defined according to criteria proposed by Liu and refers to the point along the ROC curve that maximizes the product of sensitivity and specificity and is one approach to identifying the value that best differentiates between foods meeting vs. not meeting the FOPL threshold. The value is only presented when the AUC > 0.5 and is not provided when no foods within that food category meet the threshold (e.g., 100% fruit juice and Nutri-Score value of A).*

**TABLE 2B:** Performance of bHNDS against Health Star Rating Nutri-Score by food category.

	bHNDS		HSR ≥ 5			N-S A		
	N	Mean (SD)	%	AUC	bHNDS cut-off point^a^	%	AUC	bHNDS cut-off point^a^
All			19.9	0.94		25.5	0.93	

Vegetables	390	13.7 (12.9)	69.4	0.96	5.95	92.6	0.96	1.24
Fruit	110	7.2 (4.0)	90.7	0.86	5.11	94.1	0.96	4.06
Nuts/seeds	77	7.7 (2.8)	37.2	0.94	8.38	30.6	0.97	8.72
100% juice	41	4.8 (3.0)	0.0	–	–	0.0	–	–
Milk and milk substitutes	84	2.2 (4.5)	17.7	0.94	4.67	0.0	–	–
Beans, peas, and legumes	63	1.6 (3.6)	75.6	0.90	0.21	99.4	0.76	0.21
Cooked grains	26	1.3 (1.9)	2.4	0.96	4.68	85.7	0.63	0.44
Breakfast cereals	148	0.5 (4.0)	15.4	0.89	4.60	24.7	0.90	3.95
Potatoes	85	−0.9 (1.7)	3.7	0.95	−0.30	1.8	0.96	0.46
Breads, rolls and tortillas	145	−0.9 (2.1)	0.0	–	–	26.2	0.94	−0.78
Cheese	62	−5.7 (1.7)	20.1	0.86	−5.04	0.0	–	–
Yogurt	31	−1.0 (2.3)	37.4	0.68	−1.49	53.6	0.70	−1.49
Processed soy products	13	−1.6 (6.2)	0.6	1.00	6.75	71.4	0.99	−1.73
Snacks	224	−2.0 (2.3)	0.2	0.99	4.31	1.8	0.95	−0.19
Meat, poultry and seafood	14	−2.2 (4.7)	0.0	–	–	22.8	0.90	1.72
Quick breads and bread products	100	−4.7 (2.3)	0.0	–	–	0.3	1.00	1.63
Mixed dishes	132	−4.8 (4.7)	6.6	0.97	0.97	23.3	0.68	−2.40
Sweets and desserts	456	−8.1 (3.3)	0.0	–	–	0.1	0.98	−0.62
Fats and oils	98	−8.4 (3.7)	2.7	0.27	–	0.0	–	–
Processed meat	102	−9.2 (2.3)	0.0	–	–	0.0	–	–
Jams, syrups and toppings	33	−11.7 (2.7)	0.0	–	–	1.1	1.00	1.19
Sweetened beverages	109	−13.7 (6.5)	0.0	–	–	0.0	–	–
Coffee and tea	58	−12.3 (5.7)	0.0	–	–	0.0	–	–
Condiments and sauces	122	−14 (10.9)	1.4	0.97	4.56	16.9	0.95	−5.34

*^a^The cut-off point is defined according to criteria proposed by Liu and refers to the point along the ROC curve that maximizes the product of sensitivity and specificity and is one approach to identifying the value that best differentiates between foods meeting vs. not meeting the FOPL threshold. The value is only presented when the AUC > 0.5 and is not provided when no foods within that food category meet the threshold (e.g., 100% fruit juice and NS A).*

However, the results were also category-specific. As expected, bHNDS scores differed by food category with the highest ratings obtained for vegetables, fruit, nuts/seeds, and 100% juice, and lower ratings were given to processed meat, sweetened beverages, sweets and desserts, and jams, syrups, and toppings. There was a high concordance between bHNDS values and an HSR of ≥ 4.5 or 5 stars. The AUC value was very high for numerous food categories, including beans, peas and legumes, nuts/seeds, cooked grains, breakfast cereals, and snacks. There was also high concordance between bHNDS values and Nutri-Score A/B or A grades; the AUC values were very high for most food categories. However, there were some differences across systems, notably in rating the healthfulness of beans, peas, and legumes.

The AUC analyses allowed us to establish those bHNDS threshold values that could be used as cut-off points to predict the number of HSR stars or Nutri-Score letter grades. Those threshold values cannot be calculated if the proportion of foods meeting the threshold is 0% but should only be considered a reliable predictor when the AUC is reasonably high (e.g., >0.80). For example, only 1.6% of snacks earned 4.5 or more HSR stars, but the AUC was high (0.95) and the identified bHNDS cut-off point was 0.89. By contrast, the proportion of breakfast cereals earning 4.5 or more HSR stars was much higher (26.3%), and so was the bHNDS cut-off point (3.95). Similarly, milk beverages score very differently on Nutri-Score as compared to HSR; in the present calculations, milk would need to be above HNDS −0.34 to score 4.5 HSR stars but would need to have bHNDS > 12 to have a favorable score with Nutri-Score. These differences address the importance of assessing the diagnostic performance of alternative NP models separately by food category.

## Discussion

The purpose of NP models is to “diagnose” or otherwise identify those foods that provide optimal nutritional value. Such foods have been variously described as healthful, wholesome, nutrient-dense, or nutrient-rich (3, 4, 7, 9). Foods that are awarded A or B grades (Nutri-Score) or 4 or 5 stars (HSR) are generally viewed as conforming with dietary guidelines (29).

However, NP models do not always rate individual foods in a consistent manner (1). Existing NP models can be across-the-board, or category-specific; compensatory or not; balanced or not; and based on nutrients only, or based on some combination of nutrients, food groups, and dietary ingredients (7–9). Whereas NRF and now the bHNDS models are fully compensatory, the Nutri-Score and HSR are not. “Good” points cannot be applied once “bad” points exceed a pre-set amount. Both the NRF (12) and the French SAIN, LIM model (11) were based on nutrients only; the newer hybrid scores are based on both nutrients and food groups (9, 10). The present bHNDS can be described as an across-the-board, fully compensatory, balanced NP model that integrates both nutrients and food groups. The use of mean %DV for subscore calculation weights the bHNDS more heavily toward nutrients to limit, namely saturated fat, added sugar, and sodium.

Validating new NP models against the HEI has been the standard practice (12). The new bHNDS shows a higher correlation with HEI than the purely nutrient-based NRF—this is not surprising since the HEI is also composed of nutrients and some of the same food groups. The bHNDS elements align well with the DGA shortfall nutrients, and also with the USDA Healthy Food Patterns that include whole grains, nuts, seeds, legumes, dairy, vegetables, and fruits (5). These desirable food groups are included in the bHNDS that makes for better alignment between NP methodology and dietary guidance (2). Global dietary guidelines have stressed the importance of whole grains as a potential index of carbohydrate quality (30) and there is a new emphasis on high-quality protein from plant-based or animal sources (31).

The diagnostic accuracy of alternative tests is commonly measured using ROC methods (13, 14). In very few cases (32), those methods were applied to assess the performance of HSR as applied to milk, yogurt, and cheese. One test of the diagnostic accuracy of the HSR system (32) showed that the optimal ROC cut-off point for dairy beverages corresponded to a rating of four HSR stars. However, the HSR had no discriminating power for predicting the nutrient density of yogurt or cheese. Improving methods to assess the nutrient density of foods across diverse food groups remains a priority for global public health. The present analyses show which bHNDS cut-off points were predictive of desirable high ratings on Nutri-Score and the HSR. However, the cut-off points were highly category specific.

The use of ROC analyses to test the diagnostic accuracy of alternative NP models remains relatively novel. This approach should be added to the toolbox as it allows researchers to determine the extent of agreement between two (or more) alternative NP systems. It is worth noting that Labonté et al. (1) identified a total of 387 potential NP models, including 361 from the full-text assessment of >600 publications. As NP models proliferate, it becomes important to identify potential cut-off points or thresholds that could be used moving forward. It is also important to keep in mind that different food groups have different nutritional profiles so the application of the same across-the-board NP scores across all foods and/or beverages may be inappropriate. The current trend in NP methodology is toward scores that are category-specific and more appropriate for use as benchmarks guiding product reformulation by the food industry. The one study that examined the performance of HSR relative to the NOVA classification for dairy products showed that the results were category-specific (32).

This analysis showed strong associations between bHNDS, HSR, and Nutri-Score. This was for the entire FNDDS and the associations also held within most, but not all, food categories assessed. We then showed the correspondence between bHNDS cut-off points and the percentage of foods that got the highest HSR or Nutri-Score ratings. In other words, bHNDS cut-off points could predict HSR five star or Nutri-Score A scores. This relation also holds within food groups. The cut-offs essentially show that we can mimic HSR or Nutri-Score using bHNDS.

Some limitations of the current research should be noted. First, the FNDDS is merely a proxy for the food supply and may not capture the complete diversity of foods. To eliminate this limitation, the foods were weighted by their frequency of consumption by NHANES participants. Second, the current methods for assessing diet quality, including the HEI-2015, assume that intakes higher than reference values are always positive. The HEI-2015 scoring system uses pre-selected maxima and higher intakes are neutral in the sense that they do not add to or detract from the final score. Very few NP models have used optimal ranges rather than maximum scores. In those models, energy and nutrient intakes that were either below or above pre-defined healthy ranges were assigned lower diet quality scores (33). Third, both Nutri-Score and the Health Star Rating essentially transform energy density. Despite their widespread use, they may not identify or “diagnose” foods that provide optimal nutritional value. Indeed, no such standard may exist in practice.

## Conclusion

The present bHNDS model is compensatory, balanced, and based on both nutrients and food groups. Analyses pointed to high correlations with two FOPL systems: Nutri-Score and HSR. ROC curve analyses by category were used to predict whether a given food would receive the A/B-grade or a 4/5-star rating. Given the multiplicity of NP schemes, regulatory agencies would benefit from cross-comparisons and some degree of harmonization. The bHNDS represents one way to help identify food providing optimal nutrition.

## Data Availability Statement

Publicly available datasets were analyzed in this study. This data can be found here: All data for this project are publicly available on the National Center for Health Statistics and United States Department of Agriculture Website, available at: https://wwwn.cdc.gov/nchs/nhanes/Default.aspx and https://www.ars.usda.gov/northeast-area/beltsville-md-bhnrc/beltsville-human-nutrition-research-center/food-surveys-research-group/docs/fped-overview/, respectively.

## Author Contributions

AD: methodology, conceptualization, writing-original draft, and writing-review and editing. TG: methodology, conceptualization, formal analysis, and writing-review and editing. CR: methodology, conceptualization, formal analysis, writing-original draft, and writing-review and editing. All authors contributed to the article and approved the submitted version.

## Author Disclaimer

The views expressed in this manuscript, however, are those of the authors and do not necessarily reflect the position or policy of PepsiCo, Inc.

## Conflict of Interest

TG and CR were salaried employees of PepsiCo, Inc. which funded this research. The funder was not involved in study design, methodology development, analysis, interpretation of data, or the writing of this article but did review the article approving it for publication. AD was the originator of the Nutrient Rich Food Index, an early NP model, and has received grants, contracts, and honoraria from entities both public and private with an interest in nutrient profiling and (re) formulation of foods. AD has served as consultant to PepsiCo, Inc. for this project.

## Publisher’s Note

All claims expressed in this article are solely those of the authors and do not necessarily represent those of their affiliated organizations, or those of the publisher, the editors and the reviewers. Any product that may be evaluated in this article, or claim that may be made by its manufacturer, is not guaranteed or endorsed by the publisher.
