# Supplementary material for: Balanced Hybrid Nutrient Density Score Compared to Nutri-Score and Health Star Rating Using Receiver Operating Characteristic Curve Analyses
Source: Front Nutr. 2022 May 2;9:867096. doi: 10.3389/fnut.2022.867096 (PMC9108770; doi:10.3389/fnut.2022.867096)
Supplement: Supplementary file 1 [file Table_1.docx]

Balanced Hybrid Nutrient Density Score (bHNDS) compared to Nutri-Score and Health Star Rating using Receiver Operating Characteristic (ROC) curve analyses

Adam Drewnowski^1*^, Tanhia D. Gonzalez^2^, Colin D. Rehm^3^

^1^Center for Public Health Nutrition, University of Washington, Seattle, WA, United States

^2^PepsiCo, Plano, TX, United States

^3^PepsiCo, Purchase, NY, United States

Supplemental Figures and Tables

**Supplemental Table 1.** Correlation coefficients between bHNDS component scores at the food-level
